# Endodontic disease detection: digital periapical radiography versus cone-beam computed tomography—a systematic review

**DOI:** 10.1117/1.JMI.8.4.041205

**Published:** 2021-02-24

**Authors:** Kehn E. Yapp, Patrick Brennan, Ernest Ekpo

**Affiliations:** The University of Sydney, School of Health Sciences, Medical Image Optimisation and Perception Group, Discipline of Medical Imaging Science, Faculty of Medicine and Health, Australia

**Keywords:** cone-beam computed tomography, diagnostic performance, periapical radiography

## Abstract

**Purpose**: To assess the comparative diagnostic performance of digital periapical (PA) radiography and cone-beam computed tomography (CBCT) imaging on endodontic disease detection and to provide study methodology and design recommendations for future studies comparing the diagnostic performance of imaging modalities on endodontic disease detection.

**Approach**: A search of the Medline, Embase, Scopus, Web of Science, and the Cochrane Central Register of Controlled Trials databases was conducted. Studies that compared the performance of CBCT to digital PA radiography for detecting endodontic disease had an independent reference standard determining the presence of endodontic disease and conducted data analysis including either sensitivity, specificity, receiver operating characteristic (ROC) analysis or free response operating characteristic analysis were included. Of the 20,530 identified studies, only 3 fulfilled the inclusion criteria.

**Results**: Most studies assessed for eligibility were excluded due to limitations and biases in study design—15 of 18 studies had no reference standard. Only one retrospective clinical study reported on the diagnostic performance of CBCT and showed a sensitivity of 86% and specificity of 26%. Two cadaver studies reported sensitivity ranging from 60% to 100%, specificity ranging from 79% to 100%, and an area under the ROC curve of 0.943 for CBCT. The reported sensitivity for digital PA radiography ranged from 27% to 60%, specificity was 99%, and the area under the ROC curve was 0.629.

**Conclusions**: There is a lack of quality evidence and insufficient data to compare diagnostic performance of digital PA and CBCT imaging. This emphasizes the need for well-designed studies to inform clinicians about the relative diagnostic performance of these imaging modalities.

## 1 Introduction

Endodontic disease prevalence has been reported to range from 7% to 86%,^1^ and it is estimated that 22 million endodontic procedures are performed annually in the United States of America.^2^ Prior to these procedures, dental imaging is required not only for diagnostic, but also for medico-legal and treatment planning purposes.^3^ Diagnosis of dental and endodontic abnormalities follows a Bayesian approach just like in medicine—patient history and examination data are gathered to generate pre-test odds (prior probability) of a disease being present. This is multiplied by the weight of new testing information (likelihood ratio) that generates post-test odds (posterior probability) of the disease being present.^4^ Dental imaging has historically used intra-oral and extra-oral diagnostic radiographs, with an early cadaver study showing the limitations of radiographs in showing simulated pathologic changes in cancellous bone; these radiolucent changes could be detected radiographically only if there was cortical bone perforation.^5^ A later clinical study showed that periapical (PA) radiography had high diagnostic value in endodontic disease detection.^6^

Medical imaging is constantly evolving; three-dimensional (3D) cone-beam computed tomography (CBCT) has been recently introduced into the clinical dental setting and is gaining popularity.^7^ Newer imaging modalities can be considered fit for purpose if their diagnostic performance is comparable to, or better than, current modalities. Diagnostic efficacy analysis is therefore needed to establish the diagnostic ability of these new imaging tools.^8^ Since the introduction of CBCT into the clinical evaluation of endodontic diseases, several studies have attempted to investigate its diagnostic efficacy compared to two-dimensional PA radiography. They showed differences in the diagnostic performance of these modalities; however, these studies differ by design and results. For example, a majority of the studies was based on imaging examination records of PA, panoramic, and CBCT imaging and included patients with different presentations of endodontic infections or patients referred to a specialist endodontic practice for endodontic treatment, with a consensus opinion of a panel being used to establish the presence of disease.^9^ Second, most of these studies did not perform an evaluation of CBCT using an established independent reference standard.^10^ Third, many of the studies were based on conventional PA radiography and either assessed the agreement between CBCT and PA reporting or PA and panoramic image reporting. Most utilized only images with disease, which limited the calculation of diagnostic performance metrics such as specificity and false positive rates. Some of the published studies used CBCT as a “reference standard” to assess the sensitivity of PA imaging. These differences in methodologies and result frameworks emphasize the need for a review of the literature to understand the diagnostic efficacy of CBCT relative to PA radiography.

Previous systematic reviews comparing CBCT and PA radiography in endodontic disease detection^11^^–^^15^ were mostly based on plain film PA radiography, included studies that assessed the agreement between both imaging modalities or used cadaver findings as a reference standard. Some used an artificial reference standard: “mechanically or chemically induced lesions,” which does not establish the truth about endodontic disease presence or absence.^12^^,^^14^ Two of these reviews, which focused on the diagnostic efficacy of CBCT and PA radiography using a hierarchical model, reported that the diagnostic efficacy was unclear^13^ and that human CBCT studies using a histological reference standard were needed.^12^ A meta-analysis of *ex-vivo* studies with artificial apical periodontitis found CBCT imaging had a greater area under the receiver operating characteristic (ROC) curve than PA radiography.^14^ A more recent review showed that the odds ratio of CBCT detecting endodontic disease was double that for PA radiography^15^. However, these reviews have some limitations: the diagnostic performance of digital PA and CBCT imaging were not compared directly,^11^^–^^13^^,^^15^ *ex-vivo* studies were included, which limit the external validity of these findings^11^^,^^12^^,^^14^ and therapeutic efficacy rather than diagnostic accuracy was evaluated.^12^^,^^13^ Therefore, the comparative diagnostic performance of these imaging modalities in the endodontic domain is poorly understood.

In addition, digitization has improved the quality of radiological images and allowed post-processing of acquired images to suit different diagnostic tasks. In dentistry, digitization of the imaging process has been shown to improve image quality, which may optimize the detection of dental caries and assessment of bony anomalies.^16^ Thus, a review of the literature on the diagnostic performance of CBCT relative to digital PA radiography in the digital era will provide informed choices of imaging options for patients and clinicians requesting dental imaging. This review aims to assess the comparative diagnostic performance of digital PA and CBCT imaging on endodontic disease detection and to provide study methodology and design recommendations for future studies comparing the diagnostic performance of imaging modalities on endodontic disease detection.

## 2 Methods

### 2.1 Databases and Search Strategy

The literature search was conducted based on the Preferred Reporting Items for a Systematic Review and Meta-Analysis of Diagnostic Testing Studies (PRISMA-DTA) statement.^17^ Medline, Embase, Scopus, Web of Science, and the Cochrane Central Register of Controlled Trials databases were searched for relevant articles published from database inception to January 12, 2021. Google Scholar was also used to search for relevant articles and the reference lists of published articles were manually screened to identify additional publications. Search terms were combined with “OR” and included the following main terms: “Cone beam computed tomography” OR “cone beam” OR “periapical radiography” OR “periapical” OR “endodontics” OR “pulp disease” OR “apical periodontitis” OR “periapical disease” OR “periapical lesion” OR “endodontic pathosis” OR “apical pathology” OR “apical radiolucency” OR “receiver operating characteristic” OR “free response.”

### 2.2 Eligibility Criteria

Inclusion and exclusion criteria were based on the population, intervention, comparator, and outcome (PICO) elements (Table 1). The clinical research question we sought to address was, in permanent human teeth, does CBCT have greater diagnostic performance in endodontic disease detection than PA radiography? Studies were included if they: compared the performance of CBCT to digital PA radiography for detecting endodontic disease, included humans with permanent teeth, had an independent reference standard determining the presence of endodontic disease, conducted data analysis including at least one of the following outcomes: sensitivity, specificity, ROC analysis, or free response operating characteristic (FROC) analysis, and were published in English. Studies were excluded if they did not meet these inclusion criteria. Literature reviews, conference papers, letters to editors, and posters were also excluded. Initial triage of the abstracts was performed by two authors (K.Y. and E.E.). Disagreements were resolved by objectively evaluating the inclusion and exclusion criteria and establishing a consensus.

**Table 1:** The PICO method regarding inclusion and exclusion criteria.

Element	Characteristics
Population	Permanent human teeth
Intervention	CBCT imaging
Comparator	Digital PA radiography
Outcome	Diagnostic performance in endodontic disease detection: ROC curve analysis, FROC analysis, sensitivity, and specificity

### 2.3 Quality Assessment

Quality assessment was performed by two authors (K.Y. and E.E.) using the Quality Assessment of Diagnostic Accuracy Studies (QUADAS-2) tool.^18^ It consists of four main domains: patient selection, index test, reference standard, and flow and timing of the index tests and reference standard. The QUADAS-2 tool is mainly recommended for judging the risk of bias and the applicability of original diagnostic accuracy studies. A weighted kappa was used to assess the agreement between the two assessors. Kappa was interpreted as follows: <0.20=poor; 0.21 to 0.40 = fair; 0.41 to 0.60 = moderate; 0.61 to 0.80 = substantial; and 0.81 to 0.99 = almost perfect.^19^ Any discrepancies in the quality assessment were discussed and resolved through consensus.

### 2.4 Data Extraction Process

Data were extracted in two phases. First, the authors determined the study characteristics (e.g., study design, reported outcome measures, provision of clinical history, and recruitment method for patients and readers), population characteristics (e.g., sample size, disease prevalence, and distribution of disease severity), reader characteristics (observer clinical experience, CBCT experience, and qualifications), and interpretation protocol. Second, the diagnostic performance of PA imaging was compared to CBCT. The performance metrics analyzed were number and location of detected abnormalities, ROC curve construction, relationship between true positive fraction (TPF) at a given false positive fraction (FPF), area under the ROC curve, FROC analysis, and diagnostic accuracy measures such as jackknife FROC figure of merit, sensitivity, and specificity. All authors reviewed the full text articles and any discrepancies regarding data analysis or interpretation were resolved by objectively evaluating the reported findings and establishing a consensus.

## 3 Results

### 3.1 Identification of Included Studies

**Fig. 1:**
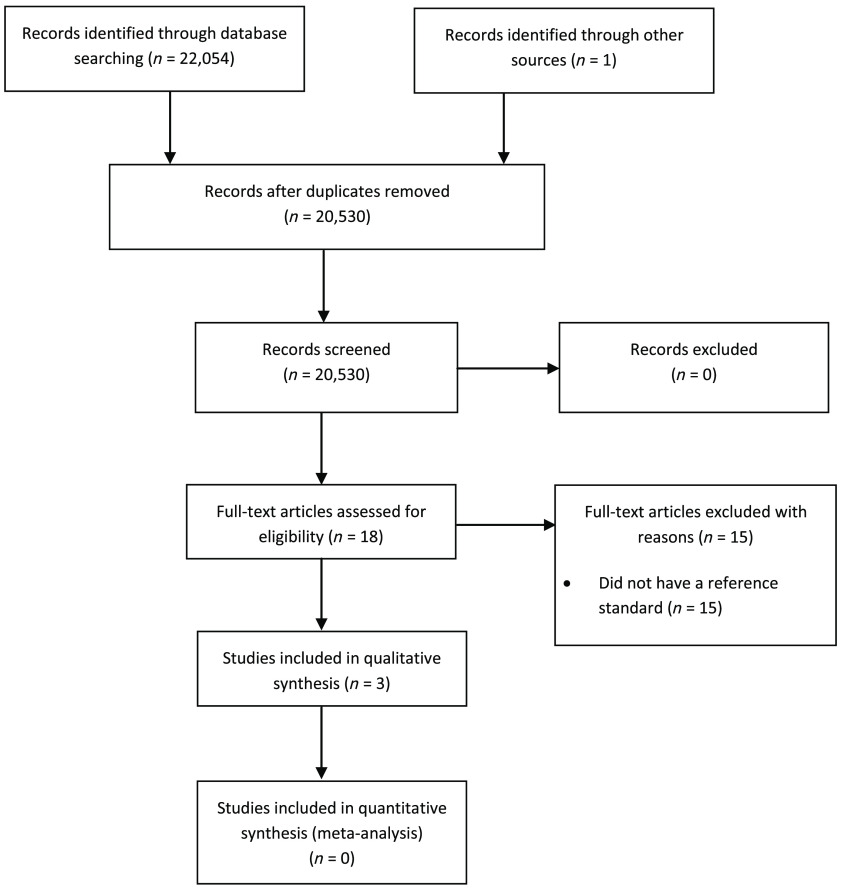
A Preferred Reporting Items for Systematic Reviews and Meta-Analysis (PRISMA) statement flowchart of the search and selection strategy.

The search strategy identified a total of 20,530 studies. After the screening of titles and abstracts, 18 studies were selected for full-text reading (Fig. 1). Only three studies fulfilled the inclusion criteria.^20^^–^^22^ One study used clinical information^20^ and two used data from cadavers^21^^,^^22^ as the reference standard to assess the diagnostic performance of CBCT and digital PA radiography (Table 2). Fifteen studies that were identified to have examined disease detection using CBCT and digital PA radiography but were excluded are summarized in Table 3. These studies were excluded for the following reasons: none had an independent reference standard, either did not provide information about disease prevalence or used only images with disease —limiting the assessment of other diagnostic performance metrics, assessed agreement between CBCT and PA radiography reports, or did not provide diagnostic performance metrics. None of these studies accounted for case difficulty and severity of diseased or non-diseased patients.

**Table 2:** Characteristics of included studies.

Study	Sample size (teeth)	Participant characteristics	Disease prevalence	Index test	Reference standard	Readers	Disease severity distribution	Performance metrics provided
Pope et al.^20^	200	Retrospective record analysis from endodontic practices	7.8% (14/180)	Diameter of PA radiolucency using modified CBCT-PA index (PAI) scale	Clinical information	Two: one endodontist and one endodontic postgraduate student	No	CBCT sensitivity (86%) and specificity (26% to 80%)
Kanagasingam et al.^21^	67	Cadavers	Not provided	Presence of a “PA lesion”	Histopathology findings	Five endodontists	No	Sensitivity, specificity, and area under the ROC curve. CBCT (89%, 100%, and 0.943), PA (27%, 99%, and 0.629)
Kruse et al.^22^	222	Cadavers	Not provided	Presence of apical periodontitis using a 5-point rating scale	Histopathology findings	Three: two endodontists and one oral radiologist	No	CBCT and PA sensitivity (80%, 60%) and CBCT specificity (79%)

**Table 3:** Characteristics of excluded studies.

Study	Sample size (teeth)	Disease prevalence	Index test	Reference standard	Readers	Performance metrics provided
Lofthag-Hansen et al.^23^	46	100%	Consensus report of a PA lesion	No	Three oral and maxillofacial radiologists	None
Estrela et al.^10^	1508	100%	PAI score	No	Three “calibrated examiners”	None
Low et al.^24^	74	100%	Consensus report of a PA lesion	No	Two: one oral radiologist and one endodontist	None
Lennon et al.^25^	10	100%	Presence of artificial bone lesions using a 5-point rating scale	No	Ten: two endodontists, two dental radiologists, and six postgraduate endodontic students	None
Abella et al.^26^	138	100%	Consensus report of an “apical periodontitis lesion”	No	Two endodontists	Number of “lesions” seen on PA and CBCT
Patel et al.^9^	151	100%	Consensus report of an apical periodontitis lesion	No	Two endodontists	Number of lesions seen on PA and CBCT
Abella et al.^27^	161	100%	Consensus report of an apical periodontitis lesion	No	Two endodontists	Number of lesions seen on PA and CBCT
Venskutonis et al.^28^	35	Not provided	Consensus report of a PA lesion	No	Two endodontists	None
Bornstein et al.^29^	58	100%	Report of either “cyst” or “granuloma”	No	Four: two oral surgeons and two oral surgery residents	Number of radiographic reports designated as granuloma or cyst
Davies et al.^30^	100	100%	Consensus report of a PA lesion	No	Two endodontists	Number of roots with a PA lesion detected
Weissman et al.^31^	67	Not provided	Presence of apical radiolucency	No	Three: two endodontists and one oral and maxillofacial radiologist	Number of lesions seen on PA and CBCT
Davies et al.^32^	98	Not provided	Consensus report on the change in PA status at review	No	Two endodontists	Healing or non-healing category
Beacham et al.^33^	18 imaging studies	Not provided	Report on the location of any finding considered “notable or important”	No	Nine: four endodontists and five endodontic residents	Number of radiographic findings assigned by an “expert reviewer” that were identified by the observer
Kruse et al.^34^	74	Not provided	Consensus score determining the level of healing and the treatment plan	No	Three: two endodontists and one oral radiologist	Change in treatment plan based on CBCT report
Chang et al.^35^	68 imaging studies	Not provided	Presence of a PA lesion	No	Two: one endodontist and one oral and maxillofacial radiologist	Number of lesions seen on PA and CBCT

### 3.2 Quality Assessment

The three included studies had a different risk of bias and a range of applicability concerns regarding patient selection and reference standard. For all three, the risk of bias about the index test was low and risk for flow and timing was uncertain. All studies had high applicability concerns about the index test. The quality assessment results are summarized in Table 4. Inter-reader agreement between the two assessors of quality showed a weighted kappa of k=0.92, 95% CI: 0.767 to 1.000.

**Table 4:** QUADAS-2 tool results.

Study	Risk of bias				Applicability concerns		
	Patient selection	Index test	Reference standard	Flow and timing	Patient selection	Index test	Reference standard
Pope et al.^20^	High	Low	Low	Uncertain	Low	High	Low
Kanagasingam et al.^21^	Uncertain	Low	Uncertain	Uncertain	Uncertain	High	Uncertain
Kruse et al.^22^	Uncertain	Low	Uncertain	Uncertain	Uncertain	High	Uncertain

### 3.3 Diagnostic Performance of Periapical Radiography versus Cone-Beam Computed Tomography

When clinical information was used as the reference standard and disease was considered to be having a PA radiolucency with diameter >0.5  mm, CBCT had a sensitivity of 86% (12/14).^20^ When non-disease was considered to be an intact PA bone structure, specificity was 26% (43/166). If the threshold for non-disease was PA radiolucency with diameter no greater than 1 mm, CBCT specificity was 80% (133/166). Although both modalities were compared with different methods of analysis, no data were reported on the diagnostic performance of PA radiography.

For cadaver studies, sensitivity of CBCT for detecting endodontic disease ranged from a mean of 89%^21^ to 80% (66/83) when individual roots were calculated with no figures provided for teeth.^22^ Sensitivity of PA radiography was reported to range between a mean of 27%^21^ and 60% (134/223) for individual roots.^22^ Specificity of CBCT varied from 79%^22^ to 100%^21^ when individual roots were calculated. PA radiography had a reported specificity mean of 99%.^21^ When ROC data were given, the area under the curve values were 0.629 for PA radiography and 0.943 for CBCT.^21^ Data on the relationship between true and false positive fractions—the sensitivity at a given specificity, and vice versa, were not provided.

The three included studies each had different index tests and displayed methodological heterogeneity in reporting measures. The cadaver histology results used the presence of inflammation as the reference standard; however, the relationship to disease in humans was not shown. Due to the methodological heterogeneity, meta-analysis was not performed.

## 4 Discussion

The analysis shows that there is a lack of high-level evidence, with notable uncertainty about the study quality and bias, regarding the diagnostic performance of digital PA radiography and CBCT for endodontic disease detection. The purpose of evaluating diagnostic performance is to determine diagnostic accuracy efficacy^8^ so that the truth in yielding an abnormal or normal diagnosis can be ascertained. Evaluation at this level is clinically relevant as it forms part of the framework in a model of understanding decision making.^8^ Without the truth value of the test being evaluated, diagnostic performance is unknown.^36^ Only one study provided sensitivity, specificity, and area under the ROC curve metrics for both modalities,^21^ using cadaver samples. Of the identified studies, there were issues with study design that limit the external validity of the available data. A major finding in studies that have examined endodontic disease detection using CBCT and PA radiography was that they were designed with the aim of observers identifying radiographic findings, such as PA radiolucency, which may not always be pathognomonic for disease.^37^ In contrast, endodontic disease such as irreversible pulpitis can occur in the absence of PA radiolucency^26^ and the presence or absence of a PA radiolucency as a surrogate measure for disease has not been shown to be a relevant or valid proxy. Instead of using these reported test indices, observers should be rating their confidence in the presence of an abnormality.^38^

Population sampling across studies on CBCT and PA radiography has been skewed to contain only diseased cases. The only retrospective clinical study that fulfilled inclusion criteria had a very low disease prevalence,^20^ with cadaver studies focusing on roots, not teeth, having unknown disease prevalence.^21^^,^^22^ The main limitation of this skewed sampling strategy is that indices of test accuracy calculated in one patient group cannot be generalized to other groups if they show different clinical spectra.^39^ It should be noted that the rationale for assessing performance of a diagnostic system using a sample of cases, observers, and readings is to provide an estimate of how the imaging system would perform “on the average” in those similar cases and observers and readings that were not studied.^40^ Therefore, it is important that a diagnostic test performance study encompasses cases with a wide distribution of clinical features and includes a broad range of patients both with and without the disease.^41^ Such inclusion criteria provide opportunities to assess other diagnostic metrics, considering the variability in population characteristics and disease conditions encountered in the clinical setting. The exclusion of patients with a specific condition or high prevalence may influence observer interaction with the images and lead to inflated diagnostic accuracy estimates,^36^ particularly when cases with presenting diagnostic difficulty are excluded.^18^ Low-quality studies with a non-representative sample have a tendency to overestimate the diagnostic performance of a test.^42^ A test-set containing a wide distribution of cases with different levels of difficulty is needed to provide representation of the variation in the clinical setting,^43^ where diagnostic performance decreases as disease findings become more subtle.^44^ All three included studies did not report on their population case spectrum and their ability to extrapolate findings to the broader population is unknown. Only one study reported on sensitivity and specificity of both modalities using histopathology findings as the reference standard^21^ and found that sensitivity was higher for CBCT compared to PA radiography (89% and 27%, respectively), with no significant change in specificity (100% and 99%, respectively). An animal study^45^ with a similar study design also found CBCT had higher sensitivity (91%) than PA imaging (77%) with no difference in specificity (both 100%). Because the disease severity in both studies was not reported, it is unknown which case types of disease or non-disease these results apply to. Future studies should include cases with a range of severity in both diseased and non-diseased patients.^44^

Intrinsic human limitations can influence the diagnostic performance of imaging modalities and there are variations in the human ability to interpret radiological images. Diagnostic accuracy efficacy is not just a function of the image, it is a joint function of the images and of an observer.^8^ Reader variability has been shown in previous endodontic studies on PA radiography^46^^,^^47^ and CBCT.^48^ Therefore, studies assessing diagnostic image performance should include a significant number of readers. The number of observers in the identified studies had a tendency to be low and most ranged from 2 to 5, with two studies having nine and ten readers, respectively. Given that every case was read once, or a consensus report was used, the number of total opinions used to establish the performance of CBCT relative to PA radiography was low. The excluded studies also suffered from low observer numbers. In other radiology domains, certain factors such as training^49^ and number of annual cases read^50^ have been shown to be associated with diagnostic performance but no study has explored how these factors affect diagnostic performance in digital PA radiography and CBCT interpretation. No information was provided on reader experience and expertise on diagnostic performance, which is of clinical relevance; diagnostic accuracy has been shown to increase with reader experience.^51^ Therefore, future studies should account for variation in reader characteristics that affect diagnostic performance. Importantly, endodontic CBCT images are interpreted by dentists and radiologists.^7^ A comparison of the diagnostic performance of these professionals and the factors that impact their performance would help provide informed strategies for improving diagnostic efficacy of dental imaging interpretation.

Across the literature on endodontic disease imaging, there is a lack of an independent and valid reference standard for assessing the performance of CBCT or PA radiography. The reference standard is needed to establish the truth about disease presence or absence and to measure sensitivity and specificity of these imaging technologies;^52^ without it, the true test results are unknown.^36^ Biopsy has been used as a reference standard in medicine;^50^ however, for endodontic disease, histology results do not have the same level of dichotomy. Inflammatory cells in the PA tissues have been shown to be present for healed teeth^53^ and while histological findings are independent, the presence of inflammation does not necessarily indicate disease presence and has not been shown to be a valid reference standard for disease. Furthermore, the use of cadaver histology is limited due to the lack of clinical evidence to corroborate histological findings in endodontics. An example of a valid and independent reference standard in radiology studies is the Delphi panel, where examination and follow-up clinical information is given to a consensus panel who collectively determine the presence or absence of disease.^54^ It should be emphasized that these panelists are not involved in the reporting of images in the study. A Delphi panel approach should be used as a reference standard for future dental diagnostic imaging studies.

Observer performance measurement in the identified studies demonstrated significant limitations. The assigned observer task was to report on a type of radiographic finding without indicating its location. This is inconsistent with previous teachings from medical radiology that an observer’s task is to not only to detect but also to locate the abnormality.^55^ When the decision task involves more than just a determination of whether the patient is diseased or non-diseased, the bivariate ROC method has significant limitations in assessing diagnostic efficacy.^56^ To inform treatment interventions on the correct tooth, the exact location of the disease is required. Therefore, inclusion of location information in dental imaging studies is important. Without location assignment on images, errors can be disguised as correct calls.^57^ This is overcome by reporting using the free response paradigm; on an image, the reader reports a “mark”—a region suspicious for abnormality, and assigns a “rating”—the corresponding confidence level.^38^ This search paradigm accounts for ambiguities that can occur unnoticed in the ROC paradigm, such as when a location-level false positive and a location-level false negative occur on the same image.^57^ In this situation, ROC analysis provides an image-level true positive for the wrong reasons; an incorrect abnormality location was reported and an abnormality missed. Without a free response analysis, these potentially significant errors are overlooked. For this reason, future dental diagnostic imaging studies should use the free response paradigm.

This review has highlighted the limitations of the current literature on assessing diagnostic performance of dental imaging modalities, identified methodological issues, and provided examples of study designs to address these limitations. The lack of sensitivity, specificity, and relationship between TPF and FPF data in published studies emphasize the need for further studies to establish the diagnostic efficacy of CBCT relative to digital PA radiography. Only three studies were included, which further highlights the need for properly designed studies comparing digital PA and CBCT imaging in endodontic disease detection. In particular, future studies need to overcome the limitations of the existing studies and avoid repeating the errors previously made, in order to provide valid and relevant data that can help improve clinical decision making. Without further research, the comparative performance of these endodontic imaging modalities and the factors that influence their diagnostic efficacy cannot be determined.

## 5 Conclusion

There is a lack of evidence to establish the diagnostic performance of digital PA radiography relative to CBCT in endodontic disease detection. Well-designed studies are required in order to inform clinicians about the diagnostic performance of commonly used digital imaging modalities in detection of endodontic disease. These should reflect the task in clinical practice, use a valid reference standard, allow for measuring multiple abnormalities per image, include localization of abnormalities, reward correct and penalize incorrect abnormality locations, and encompass the entire spectrum of disease and non-disease severity present within the study population.
